# Targeting the RNA-Binding Protein HuR Alleviates Neuroinflammation in Experimental Autoimmune Encephalomyelitis: Potential Therapy for Multiple Sclerosis

**DOI:** 10.1007/s13311-020-00958-8

**Published:** 2020-11-16

**Authors:** Vittoria Borgonetti, Maria Domenica Sanna, Laura Lucarini, Nicoletta Galeotti

**Affiliations:** Section of Pharmacology, Department of Neuroscience, Psychology, Drug Research and Child Health (NEUROFARBA), University of Florence, Viale G. Pieraccini 6, 50139 Florence, Italy

**Keywords:** multiple sclerosis, HuR, relapsing–remitting experimental autoimmune encephalomyelitis, microglia, cytokines, blood–brain barrier

## Abstract

Multiple sclerosis (MS) is a chronic autoimmune inflammatory and neurodegenerative disease of the central nervous system characterized by demyelination, axonal loss, and motor dysfunction. Activated microglia are associated with the destruction of myelin in the CNS. Activated microglia produce cytokines and proinflammatory factors, favoring neuroinflammation, myelin damage, and neuronal loss, and it is thought to be involved in the disease pathogenesis. The present study investigated the role of post-transcriptional regulation of gene expression on the neuroinflammation related to experimental autoimmune encephalomyelitis (EAE) in mice, by focusing on HuR, an RNA-binding protein involved in inflammatory and immune phenomena. Spinal cord sections of EAE mice showed an increased HuR immunostaining that was abundantly detected in the cytoplasm of activated microglia, a pattern associated with its increased activity. Intrathecal administration of an anti-HuR antisense oligonucleotide (ASO) decreased the proinflammatory activated microglia, inflammatory infiltrates, and the expression of the proinflammatory cytokines IL-1β, TNF-α, and IL-17, and inhibited the activation of the NF-κB pathway. The beneficial effect of anti-HuR ASO in EAE mice corresponded also to a decreased permeability of the blood–brain barrier. EAE mice showed a reduced spinal CD206 immunostaining that was restored by anti-HuR ASO, indicating that HuR silencing promotes a shift to the anti-inflammatory and regenerative microglia phenotype. Mice that received anti-HuR ASO exhibited improved EAE-related motor dysfunction, pain hypersensitivity, and body weight loss. Targeting HuR might represent an innovative and promising perspective to control neurological disturbances in MS patients.

**Supplementary Information:**

The online version contains supplementary material available at 10.1007/s13311-020-00958-8.

## Introduction

Multiple sclerosis (MS) is a neurodegenerative disease affecting an estimated 2.9 people per 1000 [1]. MS is considered to be a chronic inflammatory disease driven by T-cell activity [2], followed by a degenerative process resulting in loss of the CNS myelin sheath and damage to the blood–brain barrier (BBB). Current MS treatment strategies are based on anti-inflammatory drugs or immunosuppressant agents to moderate effects of immune attacks in the CNS. Although such therapies are able to decrease the relapse rate in relapsing–remitting MS, efficacy in progressive MS is limited because they fail to reverse disease progression once it occurs [3]. Hence, due to side effects, limited long-term effectiveness, and inability to reverse disease, new improved drugs are required to effectively treat MS.

Microglia, the resident immune cells of the CNS located in the brain and spinal cord, are the first line of CNS defense. Upon exposure to pathogens or neuronal injury, microglia undergo distinct morphological transformations, migrate towards the stimulus, and secrete inflammatory cytokines such as IL-1β, TNF-α, and/or IL-6 [4], favoring neuroinflammation, myelin damage, and neuronal loss. Experimental evidence indicates that microglia are key players in MS pathology [5]. Activated microglia abolishment represses the development of experimental autoimmune encephalomyelitis (EAE), an animal model of MS, by inhibiting the infiltration of inflammatory immune cells [6]. Additionally, *in vivo* imaging revealed that perivascular microglia mediate axonal damage in the spinal cord of EAE mice [7], and activated microglia are abundantly observed in the inflammatory lesions of MS patients [8]. Many of the factors that drive this proinflammatory response, such as IL-1β, IL-6, TNF-α, and IL-17, are heavily post-transcriptionally regulated through adenine- and uridine-rich elements (ARE) present in noncoding regions of the mRNAs [9, 10]. These elements govern mRNA cytoplasmic stability and translational efficiency through interactions with RNA-binding proteins (RBP) to promote gene expression upregulation [11]. Among the RBP family, HuR is involved in the maintenance of inflammation and in the proper functioning of the immune system by positively regulating the stability of many target mRNAs, such as proinflammatory cytokines [12, 13], and recent findings described the role of HuR in defining the phenotype of activated microglia in amyotrophic lateral sclerosis [14]. In addition, HuR has been implicated in neurological pathologies, including neurofibromatosis type 1, amyotrophic lateral sclerosis, spinal muscle atrophy, and paraneoplastic encephalomyelitis [13]. Based on this evidence, HuR might represent a key regulator also in MS. In this study, we hypothesized that HuR might play an important role in the post-transcriptional regulation of expression of genes associated with activated microglia in MS. We, thus, investigated the effect produced by HuR silencing in a mouse model of relapsing–remitting EAE related to progression and severity of EAE-related symptoms.

Many reports suggest that microglia play detrimental roles in MS pathology. However, in addition to the proinflammatory phenotype, to dampen neuroinflammation, microglia can adopt an anti-inflammatory phenotype associated with resolving inflammation and tissue repair [15, 16]. Increasing evidence illustrates a proremyelination activity of microglia [17]. Regulating transition from the proinflammatory to the anti-inflammatory microglia phenotype might restore homeostasis and improve functional outcomes in MS patients. The role of HuR to influence microglia polarization towards the anti-inflammatory phenotype was also investigated.

## Methods

### Animals

Female SJL mice (Charles River, Sulzfeld, Germany), aged 10 to 12 weeks at immunization, were used to induce a mouse model of relapsing–remitting experimental autoimmune encephalomyelitis (RR-EAE). Mice were randomly assigned to standard cages, with 4 to 5 animals per cage. The cages were placed in the experimental room 24 h before behavioral test for acclimatization. The animals were fed a standard laboratory diet and tap water *ad libitum* and kept at 23 ± 1 °C with a 12-h light/dark cycle, light on at 7 a.m. All tests were conducted during the light phase. Mice weighed between 14 and 16 g at immunization. The experimental protocol was carried out after approval by the Animal Care and Research Ethics Committee of the University of Florence, Italy, under license from the Italian Department of Health (641/2017-PR) and in compliance with international laws and policies (Directive 2010/63/EU of the European parliament and of the council of 22 September 2010 on the protection of animals used for scientific purposes; Guide for the Care and Use of Laboratory Animals, US National Research Council, 2011). All studies involving animals are reported in accordance with the ARRIVE guidelines for experiments involving animals [18]. All effort was taken to minimize the number of animals used and their suffering.

All animal experiments were performed and analyzed through blinded experimenters. Each mouse in the treated or control group was numbered randomly within the weight range. After being divided randomly in each group, the animals were given their permanent number in the cages. Mice were sacrificed by cervical dislocation for removal of spinal cord for *in vitro* analyses. CO_2_ was used prior to cervical dislocation. The number of animals per experiment was based on a power analysis [19], and 10 animals per group were used to have the probability of 86% that the study detects a difference between groups at a 2-sided 0.05 significance level. Sample size was calculated by G power software. For *in vitro* assays, data are the mean of 5 individual experiments.

### EAE Induction

Mice were immunized subcutaneously (s.c.) in the flanks and at the base of the tail with a total of 200 μg of PLP_139–151_ peptide (synthesized by EspiKem Srl., University of Florence, Italy) per animal emulsified in complete Freund adjuvant (CFA; Sigma, Milan, Italy) supplemented with 4 mg/ml of *Mycobacterium tuberculosis* (strain H37Ra; Difco Laboratories, Detroit, MI). Control mice received CFA without PLP_139–151_. Immediately thereafter, and again 48 h later, all mice received an intraperitoneal (i.p.) injection of 500 ng Pertussis Toxin (Sigma) in 100 μl phosphate buffer saline (PBS). General health and body weights of all mice were assessed prior to immunization and once daily thereafter in a blinded manner until the completion of the study. Locomotor coordination and nociceptive threshold were analyzed before onset and regularly during the course of the disease.

### Antisense Oligonucleotide Administration

Phosphodiester oligonucleotides (ODNs) protected from terminal phosphorothioate double substitution (capped ODNs) against possible exonuclease-mediated degradation were obtained from Tib Molbiol (Genoa, Italy). The antisense ODN (ASO) against HuR was the following: 5′- A*T*A ACC ATT AGA CAT T*G*T-3′ in which the asterisks indicate the phosphorotioate phosphate groups. The control ODN was a fully degenerate ODN (dODN): 5′-N*N*N NNN NNN NNN NNN N*N*N-3′ in which N was randomly G, or C, or A, or T. ASO and dODN were preincubated at 37 °C for 30 min with an artificial cationic lipid (13 μM DOTAP, Sigma), to enhance both uptake and stability, before administration. The experimental protocol to test the effect of ASO included 3 control groups: dODN (as control ODN), vehicle (DOTAP 13 μM), and saline.

Using the EAE model, treatments performed before immunization neutralize the effect of an artificial disease induction and do not always address the therapeutic potential of drugs for patients at risk. HuR silencing was, thus, produced starting from day 14 from immunization, corresponding to the first disease peak. To achieve the protein knockdown, mice received a single intrathecal injection every 4 days, for a total of 4 injections. The spinal cords were removed on day 30 postimmunization.

Intrathecal (i.t.) administration was performed as previously described [20]. Briefly, the animals were placed in a transparent plastic box and anesthetized with a mixture of 4% isoflurane in O_2_/N_2_O (30:70 v/v). Then, a mask was placed over the mouse’s nose and mouth, and the isoflurane concentration was lowered to 1.5 to 2% for the remainder of the procedure. The animals were then placed in a prone position. In that way, the animal’s vertebral column was flexed around the L3–L5 level, widening these intervertebral spaces. The lumbar puncture needle was introduced perpendicular to the surface through the widest intervertebral space and lowered until it came into contact with the vertebral body. Occasionally, a short flicking of the tail or of a limb was observed. Animals then recovered in their home cage for 24 h before behavioral testing.

### Western Blot Analysis

The lumbar spinal cord dorsal horn was removed 30 days after immunization, and sample homogenates were processed as described [20]. Samples were homogenized with a pestle in a homogenization buffer containing 25 mM Tris-HCl pH = 7.5, 25 mM NaCl, 5 mM EGTA, 2.5 mM EDTA, 2 mM sodium pyrophosphate (NaPP), 4 mM p-nitrophenylphosphate (PNFF), 1 mM Na_3_VO_4_, 1 mM phenylmethylsulfonyl fluoride (PMSF), 20 μg/ml leupeptin, 50 μg/ml aprotinin, and 0.1% SDS. The homogenate was centrifuged at 9000 × g for 15 min at 4 °C, the low-speed pellet was discarded. Protein concentration in the supernatant (whole cell lysate) was quantified using Bradford’s method (protein assay kit, Bio-Rad Laboratories, Milan, Italy).

Supernatants (10 μg) were separated on 10% SDS-PAGE and transferred onto nitrocellulose membranes (120 min at 100 V) using standard procedures. Membranes were blocked in PBST (PBS containing 0.1% Tween) containing 5% nonfat dry milk for 120 min. Following washings, blots were incubated overnight at 4 °C with specific antibodies against HuR (1:1000; sc-5261; RRID:AB_627770), CD4 (1:1000; sc-19,641; RRID:AB_2009035), IκBα (1:1000; sc-1643, RRID:AB_627772), anti-p-NFκB p65, phosphorylated on Ser536 (1:500, sc-136548, RRID:AB_10610391) (Santa Cruz Biotechnology Inc., Santa Cruz, CA), CD206 (1:500; ab64693; RRID:AB_1523910) (Abcam, Cambridge, UK). After being washed with PBS containing 0.1% Tween, the nitrocellulose membrane was incubated with goat anti-rabbit horseradish peroxidase-conjugated secondary antisera (1:10,000) and left for 1 h at room temperature. Blots were then extensively washed and developed using an enhanced chemiluminescence detection system (Pierce, Milan, Italy) and signal intensity (pixels/mm^2^) quantified (ImageJ, NIH, Bethesda, MD). The exposition and developing time used was standardized for all the blots. Measurements in control samples were assigned a relative value of 100%. Several reports suggest that commonly used housekeeping proteins are not equally expressed across cell types and experimental conditions and quantification normalization of signal intensity to total protein loading is preferred [21]. For each sample, the signal intensity was normalized to that of total protein stained by Ponceau S and the acquired images were quantified using Image Lab software. Measurements in control samples were assigned a relative value of 100%.

### Immunofluorescence

The lumbar spinal cords of control and EAE mice, collected on day 30 postimmunization, were immunostained as described [20]. On day 30 postimmunization, the animals were perfused transcardially with 4% paraformaldehyde in 0.1 M phosphate buffer (PBS, pH 7.4) and the lumbar spinal cord was removed. Tissue was postfixed for 18 h with 4% paraformaldehyde at 4 °C, and transferred to 10%, then 20%, and then 30% sucrose solution. The samples were embedded in OCT, cut with a cryostat (14 μm), and thaw-mounted onto glass slides. After preincubation in 5 mg/ml BSA/0.3% Triton X-100/PBS, sections were incubated overnight at 4 °C with the primary antibody, at optimized working dilution, against HuR (1:100; sc-526; RR AB_627770), GFAP (1:100; sc-33673; RRID:AB_627673), IBA1 (1:100; sc-32725; RRID:AB_667733), IκBα (1:100; sc-1643; AB_627772) (Santa Cruz Biotechnology Inc.), CD11b (1:100; ab133357; RRID:AB_2650514), TMEM119 (1:100; ab209064; RRID:AB_2800343), and CD206 (1:100; ab64693; RRID:AB_1523910) (Abcam). After rinsing in PBS containing 0.01% Triton X-100, sections were incubated in secondary antibodies labeled with Alexa Fluor (1:400; Invitrogen, Carlsbad, CA) at room temperature for 2 h. Sections were coverslipped using Vectorshield mounting medium with 4′,6-diamidino-2-phenylindole (DAPI; Vector Laboratories, Burlingame, CA). A Leica DF 350 FX microscope with appropriate excitation and emission filters for each fluorophore was used to acquire representative images. Images were acquired with × 10 to × 20 objectives using a digital camera. Immunoreactivity was analyzed on microphotographs taken by measuring the integrated density of a region of interest after defining a threshold for background correction. The region of interest had an area of 0.05 mm^2^ and was placed on the gray matter and on the white matter. Seven spinal cord sections (separated 200 μm) of each animal were used. Measurements were performed with Fiji software (distributed by ImageJ). Colocalization of 2 different labels in the dorsal horn was measured using EzColocalization plugin (ImageJ). The extent of colocalization was determined by calculating Mander’s overlap coefficient (MOC), characterized by a range of values between 0 (complete anticolocalization) and 1 (complete colocalization).

### Hematoxylin and Eosin Staining

Mice were perfused with 4% neutral buffered formalin. Longitudinal, 8-μm sections were cut and mounted on charged glass slides. The slides were stained with 5% hematoxylin and 1% eosin (H&E), and inflammation scores were averaged from both sections. The slides were incubated in hematoxylin (5 min). Water was run over the slides for 5 min before 30-s incubation in eosin. After a tap water rinse, the slides were dehydrated by serial incubation in increasing ethanol and then xylene before being coverslipped with Permount mounting medium (Thermo Fisher Scientific, Waltham, MA). The slides were viewed on a Nikon (Tokyo, Japan) Eclipse E200 and imaged with a Nikon DS-Fi1 camera with NIS Elements software, and 10X images were merged in Adobe Photoshop 8.0 (San Jose, CA). Spinal cord pictures were graded for inflammation by a blinded investigator according to the following scale: 0 = absent or minimal infiltrates, 1 = moderate infiltrates throughout, 2 = moderate infiltrates with severe areas, and 3 = severe infiltrates throughout.

### Determination of TNF-α, IL-1β, and IL-17 from Spinal Cord

The levels of proinflammatory cytokines TNF-α, IL-1β, and IL-17 were measured on aliquots (100 μl) of spinal cord homogenate supernatants by using the Murine TNF-α, IL-1β, and IL-17A Mini ABTS ELISA Development Kits (#900-M54, #900-M47, and #900-M392, Peprotech, London, UK), following the protocol provided by the manufacturer. Briefly, frozen spinal cord samples were homogenized in 200 μl of lysis buffer (#9803 Cell Signaling Technologies, Danvers, MA) containing Protease Inhibitors Cocktail (#S8830, Sigma-Aldrich, St. Louis, MO) and 1 mM PMSF (Sigma-Aldrich) with UltraTurrax T25 (IKA Labortechnik, Staufen, Germany), followed by a 30-min centrifugation step at 10,000 ´ *g* and 4 °C. The supernatant was transferred in a clean vial and used for further determinations. Each sample was repeated in duplicate (220 μg of total protein for each sample). Values are expressed as picograms per microgram of total proteins determined over an albumin standard curve.

### Determination of TNF-α, IL-1β, and IL-17 from Plasma

Mice were put under general anesthesia by intraperitoneal injection of zoletil and xylazine cocktail. Blood samples were taken from the ventricle with a standard cardiac puncture method [22], centrifuged at 3000 × g for 10 min at 4 °C, and the plasma was collected. TNF-α, IL-1β, and IL-17 protein levels were evaluated by using noncompetitive sandwich ELISA (Biolegend e-Bioscience DX Diagnostic, Monza, Italy), following the supplier instructions. The absorbance was measured at 450 nm using a MP96 microplate reader spectrophotometer (Safas, Monaco), and cytokine levels were expressed as picograms per milliliter according to a standard calibration curve.

### Evaluation of BBB Disruption

The level of BBB disruption was detected by quantitative measurement for Evans blue content, as previously described [23], at day 30 after immunization. Briefly, mice were intraperitoneally injected with 2% Evans blue solution at a dose of 5.0 ml/kg per mouse. The dye was allowed to circulate for 4 h, and the mice were subsequently anesthetized and perfused transcardially with saline to remove the Evans blue dye in the vascular system. The brain and lumbar spinal cord were immediately removed, and images were captured. Tissues were homogenized with 2.5 ml PBS and mixed with 2.5 ml 50% trichloroacetic acid to precipitate protein overnight at 4 °C. The samples were centrifuged for 30 min at 10,000 × g, and the supernatants were measured at 610 nm for the absorbance of Evans blue by using a MP96 spectrophotometer (Safas, Monaco). The Evans blue content was expressed as micrograms per gram of brain and lumbar spinal cord.

### Behavioral Testing

Animals were habituated to the testing environment daily for at least 2 days before baseline testing and randomly assigned to each treatment group. EAE mice were monitored 3, 7, 10, 14, 18, 20, 25, 28, and 30 days after immunization. All testing was performed with a blind procedure.

#### Clinical Disease Score

Clinical disease scoring of EAE and sham mice (control group) was undertaken once daily in a blinded manner to evaluate the severity and extent of motor function deficits using a 5-point scale with half-point gradations [24]. EAE scores were daily assessed: score 0, no obvious changes in motor functions; score 0.5, distal paralysis of the tail; score 1, complete tail paralysis; score 1.5, mild paresis of 1 or both hind legs; score 2, severe paresis of hind legs; score 2.5, complete paralysis of 1 hind leg; score 3, complete paralysis of both hind legs; and score 3.5, complete paralysis of hind legs and paresis of 1 front leg. EAE clinical disease was classified as present by clinical scores ≥ 1, whereas clinical scores ≤ 0.5 were regarded as disease remission or absence. Mice reaching a score of 3.5 were excluded from the study.

#### Rotarod Test

The apparatus consisted of a base platform and a rotating rod with a diameter of 3 cm and a nonslippery surface. The rod was placed at a height of 15 cm from the base. The rod, 30 cm in length, was divided into 5 equal sections by 6 disks. Thus, up to 5 mice were tested simultaneously on the apparatus, with a rod-rotating speed of 16 rpm. The integrity of motor coordination was assessed on the basis of the number of falls from the rod in 30 s, as described [25]. The performance time was measured before and regularly after immunization.

#### Mechanical Allodynia

Mechanical allodynia was measured by using a Dynamic Plantar Aesthesiometer (Ugo Basile, Bologna, Italy), as described [26]. The mice were placed in individual Plexiglas cubicles (10 (d) x 10 (w) x 14 (h) cm on a wire mesh platform and allowed to acclimate for approximately 1 h, during which exploratory and grooming activity ended. After that, the mechanical stimulus was delivered to the plantar surface of the hind paw of the mouse from below the floor of the test chamber by an automated testing device. A steel rod (2 mm) was pushed with an electronic ascending force (0-5 g in 35 s). When the animal withdrew its hind paw, the mechanical stimulus was automatically withdrawn, and the force recorded to the nearest 0.1 g. Nociceptive response for mechanical sensitivity was expressed as mechanical paw withdrawal threshold (PWT) in grams. PWT was quantified by an observer blinded to the treatment.

The mean PWT was calculated from 6 consecutive trials (each performed every 30 min) and averaged for each group of mice before and after immunization.

#### Hot Plate Test

The hot plate test was performed as previously described [26]. Mice were placed inside a stainless-steel container, which was set thermostatically at 52.5 ± 0.1 °C in a precision water bath. The hot plate apparatus (Ugo Basile Biological Research Apparatus, Varese, Italy) was 25 × 37 × 47 (h) cm. Reaction times (s) were measured with a stopwatch. The endpoint used was the licking of the fore or hind paws. An arbitrary cut-off time of 45 s was adopted. The thermal threshold was evaluated before and after immunization.

### Statistical Analysis

All experimental results are given as the mean ± SEM. GraphPad Prism 5 was used for statistical analysis and for production of graphic representations of the data. Repeated-measures 2-way analysis of variance (ANOVA) followed by the Bonferroni test was used to compare locomotor behavior, pain behaviors, and body weights between EAE and sham mice. The Kruskal–Wallis nonparametric test followed by Dunn’s test was used to compare score values between EAE mice administered ASO, dODN, or vehicle. Score value comparison between EAE and control mice was assessed using the nonparametric Wilcoxon test. One-way ANOVA followed by the Tukey *post hoc* test was used to compare remaining data. The statistical significance criterion was *p* < 0.05.

## Results

### HuR Protein Overexpression in the Spinal Cord of EAE Mice

**Fig. 1:**
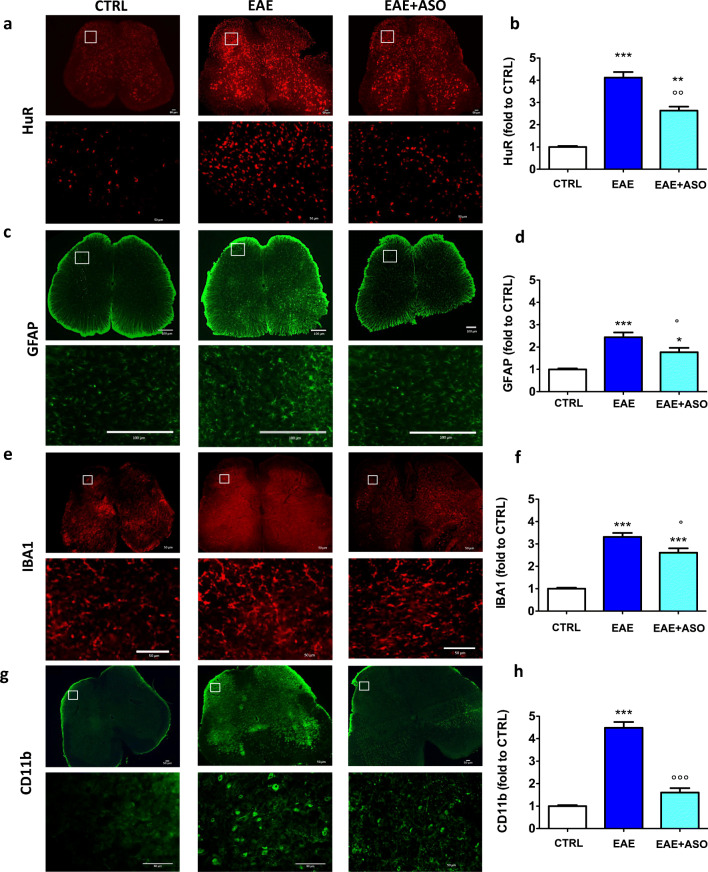
Increased expression of HuR and glial cells in spinal cords of EAE mice. (a) Representative images (low and high magnification) showing immunofluorescence staining for HuR in the spinal cords of control (CTRL), EAE, or anti-HuR ASO-treated EAE mice at 30 days postimmunization. (b) Quantification of the increased HuR immunostaining in spinal cords and significant reduction by anti-HuR ASO. Representative images (low and high magnification) show the immunostaining for GFAP (c), IBA1 (e), and CD11b (f) in the spinal cord of control (CTRL), EAE, or EAE + anti-HuR ASO mice at 30 days postimmunization. Quantification of the GFAP (e), IBA1 (f), and CD11b (h) content showing the increased expression in the EAE section and attenuation by anti-HuR ASO. CTRL, dODN-treated nonimmunized mice; EAE, dODN-treated EAE mice; EAE + ASO, anti-HuR ASO-treated EAE mice. **p < 0.01, ***p < 0.001 *versus* CTRL; °*p* < 0.05, °°°*p* < 0.001 *versus* EAE (ANOVA). Error bars represent the standard error of the mean. Antibodies are shown on the left. Scale bar = 50 μm (HuR, IBA1, CD11b); 100 μm (GFAP)

Immunofluorescence experiments were performed on spinal cord slices collected on day 30 after immunization with PLP_139–151_. Microphotographs showed the presence of a homogeneous HuR immunostaining in nonimmunized control spinal cord samples (CTRL) that was increased in PLP-EAE mice. An antisense strategy was used to investigate the role of HuR in EAE-related symptoms. HuR silencing was obtained through the repeated intrathecal administration of a specific and selective anti-HuR ASO. A dODN of the same length and chemical structure was used as control ODN. Microphotographs showed the reduction of HuR immunolabeling following treatment with an anti-HuR ASO (Fig. 1A). Quantification analysis confirmed the increase of HuR immunostaining in EAE mice, as well as the significant decrease of HuR protein expression by HuR silencing, showing the efficacy of the antisense treatment (Fig. 1B). No difference was observed among dODN-, vehicle-, and saline-treated mice, ruling out the presence of sequence-independent effects.

### Anti-HuR ASO Treatment Reduces Activated Microglia

Reactive gliosis is a common feature in MS. Studies using EAE have revealed that microglia and astrocytes actively participate in the pathogenesis of EAE progression [27, 28]. The role of HuR on glial cell activation was, therefore, investigated. We used immunofluorescence to detect HuR expression in glial cells from spinal cord slices of EAE mice. Immunization with PLP induced an increased immunostaining of the astrocyte marker GFAP (Fig. 1C) and of the microglia marker IBA1 (Fig. 1E) that were both attenuated by anti-HuR ASO administration. IBA1 staining detects both quiescent and reactive microglia and also infiltrated macrophages. To evaluate the role of HuR on activated microglia, we investigated the immunostaining of CD11b. In the lumbar spinal cord of PLP-EAE mice, we observed an abundant number of cells with intense CD11b immunoreactivity, consistent with activated microglia, which was largely reduced by HuR knockdown (Fig. 1G). Quantification analysis showed that the GFAP immunoreactivity in EAE mice exceeded that of the CTRL by 2.5-fold (Fig. 1D), the IBA1 immunoreactivity by 3.5-fold (Fig. 1F), and the CD11b immunoreactivity by 4.5-fold (Fig. 1H). A significant reduction by anti-HuR ASO of GFAP (Fig. 1D) and IBA1 (Fig. 1F) immunostaining, and a drastic reduction of CD11b immunostaining (Fig. 1H), were also confirmed.

### HuR Expression on Activated Microglia

**Fig. 2:**
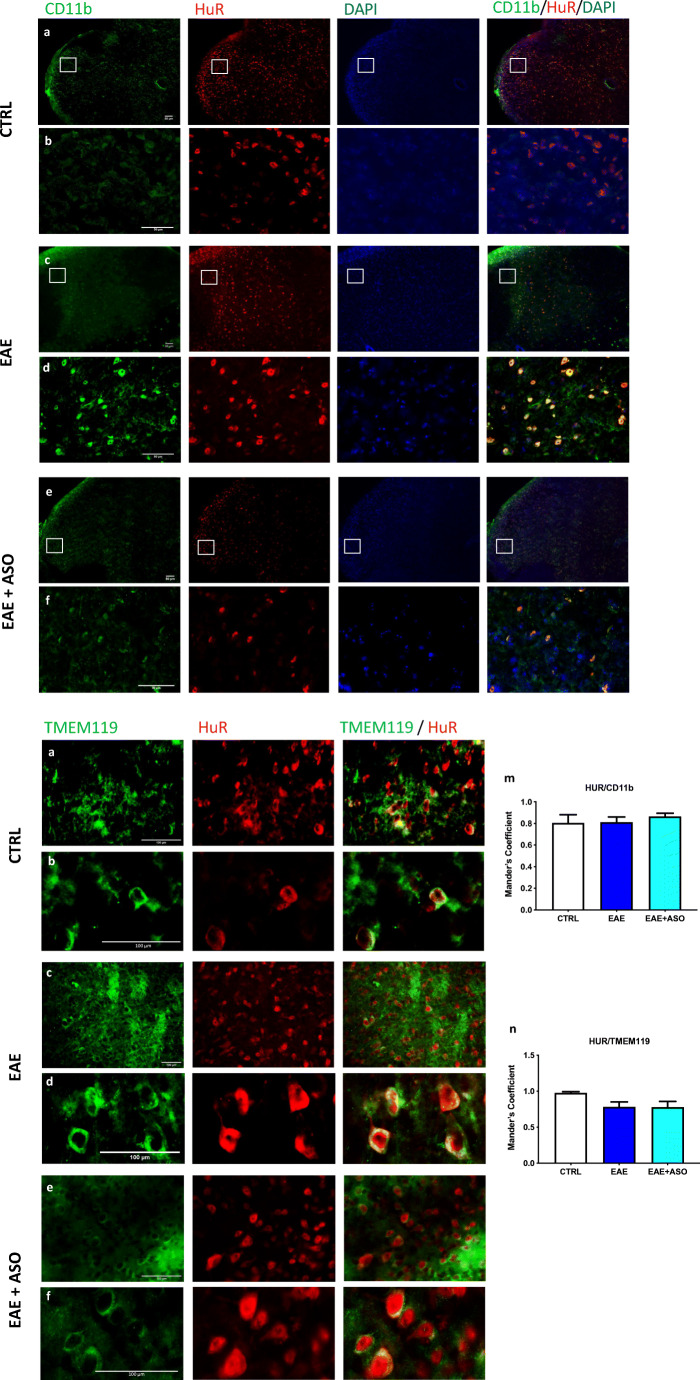
Localization of HuR protein in activated microglia from EAE spinal cord. Fluorescence microscopy images showed the microglial localization of HuR in the spinal cord of control (CTRL), EAE, or EAE + anti-HuR ASO mice. a, c, e: Low-magnification images show a homogeneous distribution of HuR within the spinal cord. Immunostaining for the microglial marker CD11b in the same section reveals similar immunoreactivity. The merged image shows HuR localization on microglial cells. b, d, f: High-magnification images show the cytosolic localization of HuR, consistent with a pattern of activation, and merged images illustrate the high degree of colocalization of HuR and CD11b. Nuclei were counterstained with DAPI. Antibodies are shown at the top. Scale bar = 50 μm. Expression of HuR protein on TMEM119-positive microglial cells. Fluorescence microscopy images showed the microglial localization of HuR in the spinal cord of control (CTRL), EAE, or EAE + anti-HuR ASO mice. g, i, k: low-magnification images. h, j, l: high-magnification images. Antibodies are shown at the top. Evaluation of colocalization of HuR and CD11 (m) or TMEM119 (n) in spinal cord sections. Scale bars = 100 μm. CTRL, dODN-treated nonimmunized mice; EAE, dODN-treated EAE mice; EAE + ASO, anti-HuR ASO-treated EAE mice

A diffuse activation of microglia is detected in the spinal cord of PLP-EAE mice on day 30 postimmunization (Fig. 2A, higher magnification in Fig. 2B) in comparison with nonimmunized control (CTRL) mice. Spinal cord sections also showed a diffuse HuR immunoreactivity in CTRL mice that was homogeneously increased in EAE mice (Fig. 2A), where localized prominently in the cytoplasm (Fig. 2B), a pattern associated with its increased activity. In most of the EAE microglia, there was a prominent merging of HuR and CD11b signals, with a pattern of cytoplasm colocalization. A high degree of expression of HuR in CD11b-positive cells was further supported by evaluation of colocalization (Mander’s coefficient, Fig. 2M).

Because macrophages and microglia will both express CD11b, to distinguish microglial cells from macrophages, the TMEM119 staining was used (Fig. 2G–L). A high degree of expression of HuR in TMEM119-expressing cells (Mander’s coefficient, Fig. 2N) confirmed the expression of HuR on microglial cells.

### Effect of Anti-HuR ASO on Inflammatory Cell Infiltration

**Fig. 3:**
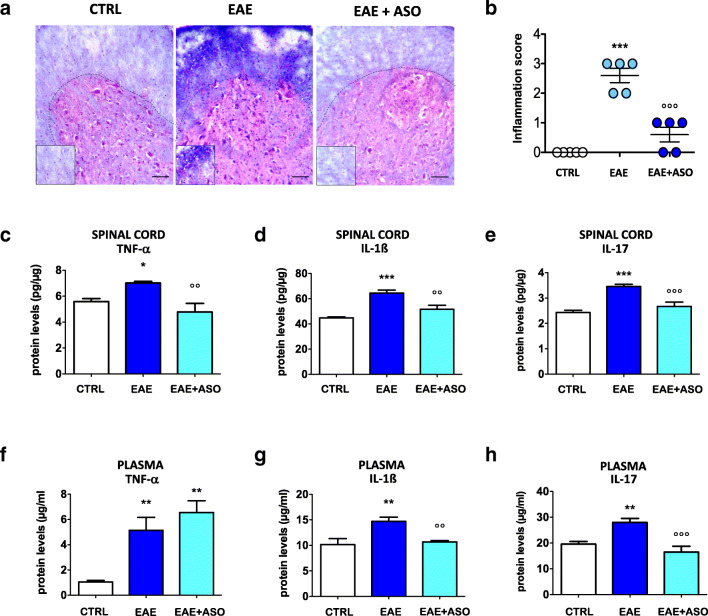
Anti-HuR ASO attenuates neuroinflammation. (a) Representative images showing spinal cord sections stained with hematoxylin and eosin (H&E) in control (CTRL), EAE, or EAE + anti-HuR ASO mice at 30 days postimmunization (high magnification on bottom images). Scale bar = 100 μm. (b) Quantification of inflammatory infiltrates indicates the large increase in their number in EAE and the reduction produced by anti-HuR treatment. Levels of TNF-α (c), IL-1β (d), and IL-17 (e) were increased in EAE spinal cords and reduced by anti-HuR ASO. Similarly, plasma levels of TNF-α (f), IL-1β (g), and IL-17 (h) were increased in EAE mice. Anti-HuR treatment reduced IL-1β and IL-17 levels and did not produce any significant modification on TNF-α levels. CTRL, dODN-treated nonimmunized mice; EAE, dODN-treated EAE mice; EAE + ASO, anti-HuR ASO-treated EAE mice. Error bars represent the standard error of the mean. *p < 0.05, **p < 0.01, ***p < 0.001 *versus* CTRL mice; °°*p* < 0.001, °°°*p* < 0.01 *versus* EAE mice (ANOVA)

To investigate whether the reduction of activated microglia produced by HuR silencing was related to a neuroinflammation dampening, we evaluated the effect of anti-HuR ASO on inflammatory cell infiltration by hematoxylin and eosin (H&E) staining of lumbar spinal cord sections. There were scarcely any inflammatory cells in samples from CTRL mice. H&E staining demonstrated a large number of inflammatory cells in PLP-EAE sections, which represented the successful induction of the disease. Inflammatory infiltration in the anti-HuR ASO-treated group was reduced in comparison with that in the PLP-EAE group (Fig. 3A, B). No significant differences in inflammatory cell infiltration were observed between nonimmunized (CTRL) and anti-HuR recipients, attesting to the remarkable anti-inflammatory effects of HuR silencing (Fig. 3B).

### Anti-HuR ASO Attenuates Proinflammatory Cytokine Expression

Because the histological examination of the PLP-immunized SJL mice revealed remarkable inflammatory cell infiltration in the spinal cord, we investigated the protein expression levels of the main proinflammatory cytokines involved in EAE, TNF-α (Fig. 3C), IL-1β (Fig. 3D), and IL-17 (Fig. 3E) in the spinal cord of PLP-EAE mice. Analysis of data showed increased secretion of all of the 3 cytokines. Treatment with anti-HuR ASO drastically reduced the content of IL-1β, IL-17, and TNF-α returning to basal levels. The plasma level of cytokines was also analyzed to determine whether the changes observed in the spinal cord were associated with the changes in the peripheral blood. The expression of the investigated cytokines was also increased in the plasma of EAE mice in comparison with nonimmunized control mice (Fig. 3F–H). Anti-HuR ASO treatment reduced the expression of IL-1β (Fig. 3G) and IL-17 (Fig. 3H), but it did not produce any effect on TNF-α level (Fig. 3F).

### NF-κB Activation Was Reduced by HuR Silencing

**Fig. 4:**
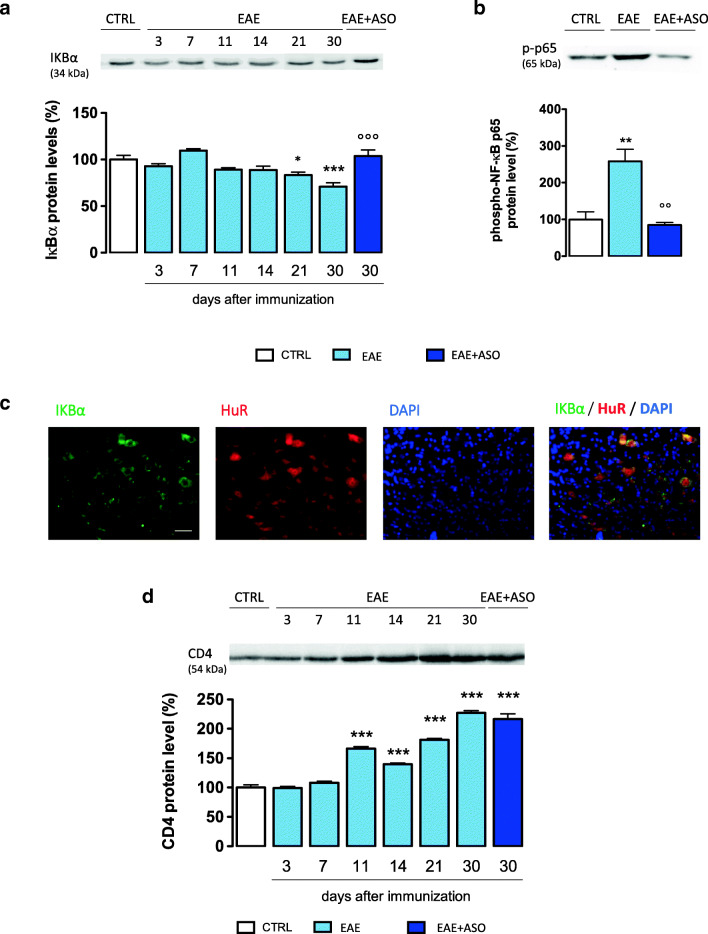
HuR silencing reduces the NF-κB signaling activation. (a) Time course evaluation showed the progressive reduction of the NF-κB inhibitory subunit IκBα. Anti-HuR ASO treatment counteracted the decrease of IκBα expression (day 30 postimmunization). (b) increased expression of the phospho NF-κB p65 subunit in the EAE spinal cord sample that is reversed by anti-HuR ASO treatment. (c) Microphotographs showed the expression of HuR on IκBα-expressing cells. Nuclei were counterstained with DAPI. Antibodies are shown at the top. Scale bars = 50 μm. (d) Time course evaluation of CD4 protein expression in spinal cord lysate of EAE mice. A significant increase in the CD4 expression was detected, and anti-HuR ASO treatment did not modify the CD4 levels on day 30 postimmunization. CTRL, dODN-treated nonimmunized mice; EAE, dODN-treated EAE mice; EAE + ASO, anti-HuR ASO-treated EAE mice. Error bars represent the standard error of the mean. *p<0.05, **p < 0.01, ***p < 0.001 *versus* CTRL mice; °°*p* < 0.01, °°°*p* < 0.001 *versus* EAE mice (ANOVA)

TNF-α and IL-17, produced by T helper 1 cells and T helper 17 cells, respectively, can activate the NF-κB pathway [29], a signaling pathway involved in neuroinflammation and implicated in the pathogenesis of MS and EAE [30]. To investigate the role of HuR in transcriptional mechanisms that promote neuroinflammation in EAE mice, the activation of NF-κB signaling was examined by immunoblotting experiments. Under basal conditions, NF-κB is inhibited by the inhibitory subunit IκBα. Following phosphorylation and degradation of IκBα, NF-κB is released and translocates to the nucleus, promoting gene transcription. PLP-EAE mice showed a progressive trend to a reduction of IκBα protein levels, indicating the degradation of the NF-κB inhibitory subunit, which became significant from day 21 postimmunization. Anti-HuR ASO treatment restored IκBα levels, thus preventing the activation of the NF-κB pathway (Fig. 4A). The activation of the NF-κB signaling is also confirmed by the staining of the phosphorylated p65 subunit of NF-κB that showed a significant increase of p-p65 expression in the spinal cord of EAE mice that was completely prevented by anti-HuR ASO treatment (Fig. 4B). Immunofluorescence photomicrographs of spinal cord slices showed the cytosolic localization of IκBα protein. Double staining immunofluorescence images showed the expression of HuR in IκBα-expressing cells, indicating that both proteins are coexpressed in the same cellular population (Mander’s coefficient, 0.832 ± 0.07; Fig. 4C).

### Lack of Anti-HuR ASO Effect on CD4 Expression

Among CD4^+^ T lymphocytes, Th1, Th17, and granulocyte–macrophage colony-stimulating factor (GM-CSF)-producing CD4^+^ T cells have been identified as important mediators in the immunopathogenesis of EAE and all of them can induce EAE independently. Because IL-17 is preferentially expressed by CD4^+^ T cells [31], the reduction by anti-HuR ASO of spinal IL-17 expression encouraged us to investigate the role of HuR on CD4 expression. We observed a progressive increase in the expression of CD4 in the spinal cord of EAE mice with peaks at day 11, day 21, and day 30 postimmunization (Fig. 4D). However, HuR silencing was unable to reduce CD4 expression (Fig. 4D). Even though HuR is involved in the maintaining of the proper function of the immune system [32], present data indicate that in PLP-EAE mice HuR has a prominent role at cytokine expression level rather than having a modulating activity of the immune response.

### Anti-HuR ASO Inhibits BBB Permeability During EAE

**Fig. 5:**
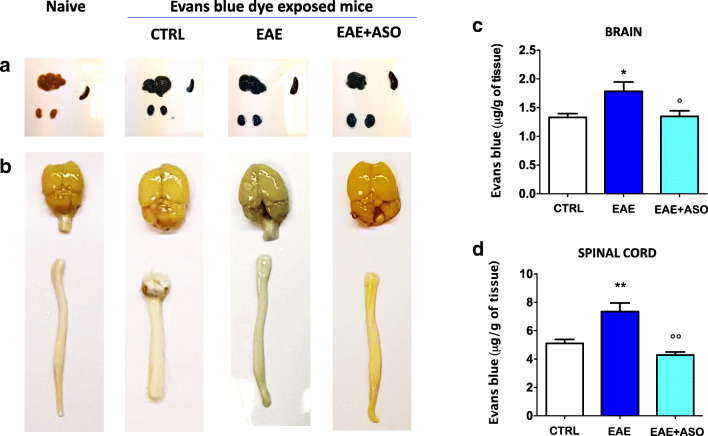
Anti-HuR ASO treatment inhibition of BBB permeability in the brain and spinal cord of EAE mice. Representative pictures of liver, kidney, spleen (a), brain, and spinal cords (b) from naïve, CTRL, EAE, and anti-HuR ASO-treated EAE mice are displayed. Blue color indicates extravasated Evans blue dye from blood vessels. The amount of extravasated Evans blue dye was quantified in the brain (c) and lumbar spinal cord (d), at 30 days after immunization (*n* = 7 per group). A significant increase of extravasated Evans blue dye was detected in the brain and spinal cord of EAE that was attenuated by anti-HuR ASO. CTRL, dODN-treated nonimmunized mice; EAE, dODN-treated EAE mice; EAE + ASO, anti-HuR ASO-treated EAE mice. Error bars represent the standard error of the mean. *p < 0.05, **p < 0.01 *versus* CTRL; °*p* < 0.05, °°*p* < 0.01 *versus* EAE (ANOVA)

Dysfunction of the BBB is one of the major features in the progression of EAE and MS. Evans Blue (EB) dye is widely used to detect a leaky BBB because it conjugates with serum albumin to form a large molecular complex which is unable to cross an intact BBB under normal circumstances. Thus, any entry of EB dye into the brain is considered an indicator of disrupted BBB permeability [33]. Consequently, we examined the EAE-induced BBB disruption by EB leakage in mouse brain and spinal cord on day 30 to determine the effect of anti-HuR ASO on BBB integrity. The macroscopic images of peripheral tissues (spleen, liver, kidney) showed a comparable exposure to EB in all groups in comparison with naïve untreated mice (Supplementary Fig. S1A). The brain and spinal cord images showed that the degree of EB leakage was increased in EAE mice (Supplementary Fig. S1B). Quantification analysis showed that the EAE group had significantly higher levels of EB extravasation than the control group in both brain (Fig. 5A) and spinal cord (Fig. 5B). The effect produced by anti-HuR ASO was evaluated. HuR silencing largely decreased the EB content in both brain and spinal cord, returning to values comparable with the baseline level of the control mice (Fig. 5A, B).

### HuR Silencing Shifted Activated Microglia from Proinflammatory to Anti-inflammatory Phenotype

**Fig. 6:**
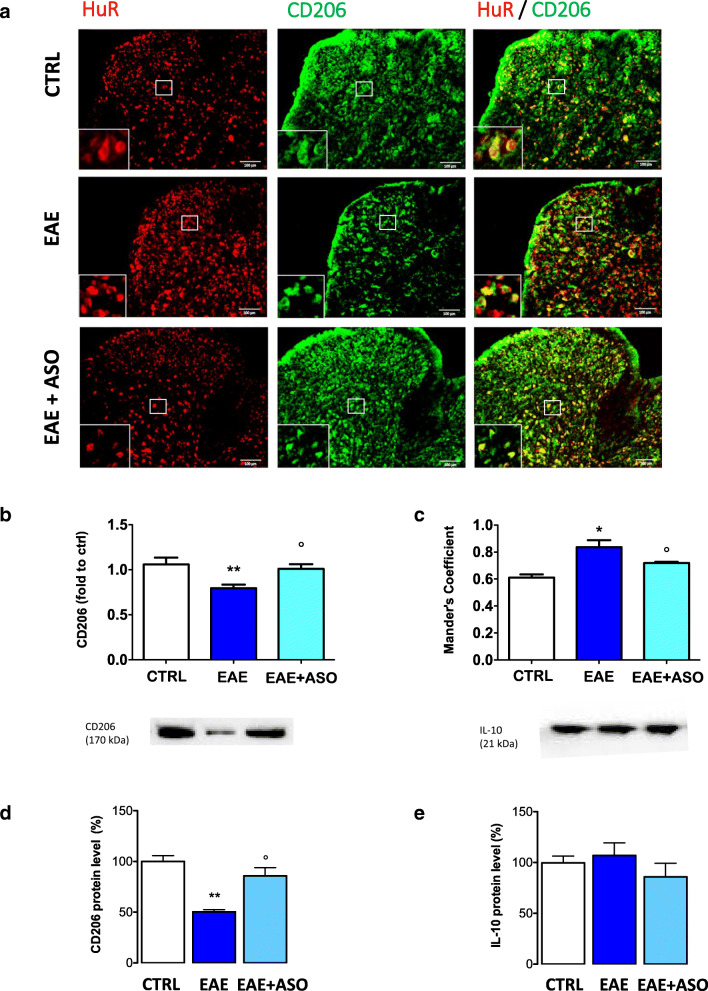
Anti-HuR ASO activated the anti-inflammatory microglial phenotype. (a) Representative microphotographs showed a decreased CD206 immunostaining in the spinal cord section of EAE mice on day 30 postimmunization. The CD206 protein expression was restored by HuR silencing. Merged images showed the expression of HuR on CD206-expressing cells. Quantification analysis of immunofluorescence (b) data showed the significant reduction of CD206 immunostaining, reversed by HuR silencing. (c) Evaluation of colocalization of HuR and CD206 in spinal cord sections. (d) Western blot results showing anti-HuR ASO reversal of EAE-induced reduction of CD206 protein expression. (e) Lack of variation of IL-10 levels in EAE mice in the absence or in the presence of HuR silencing (Western blot). (f) Representative blots of Western blotting experiments. CTRL, dODN-treated nonimmunized mice; EAE, dODN-treated EAE mice; EAE + ASO, anti-HuR ASO-treated EAE mice. Error bars represent the standard error of the mean. Antibodies are shown at the top. Scale bar = 100 μm. *p < 0.05, **p < 0.01 *versus* CTRL; °*p* < 0.05 *versus* EAE (ANOVA)

Microglia have been categorized according to their gene expression into 2 different phenotypes: microglia in proinflammatory states, referred to as proinflammatory phenotype, and microglia with anti-inflammatory phenotypes [34], and these 2 phenotypes can be detected by means of specific markers. Our results showed that anti-HuR ASO can reduce the levels of proinflammatory cytokines and reduce activated microglia. These observations prompted us to explore whether HuR might also modulate the microglial anti-inflammatory phenotype. Microphotographs from immunofluorescence experiments performed on spinal cord samples of EAE mice showed a decrease of the anti-inflammatory microglial marker CD206 that was restored by HuR silencing (Fig. 6A). The anti-HuR positive effect was confirmed by quantification analysis (Fig. 6B). Merged images showed a high degree of expression of HuR in CD206-positive cells (Fig. 6A). We also quantitated the number of microglia with high intensity merged HuR and CD206 signal, showing a high coefficient of colocalization. Results showed a significant increase in positive cells for EAE *versus* CTRL microglia (Fig. 6C). These findings are consistent with an increase of HuR in the cytoplasm of activated microglia in EAE spinal cords. A decrease of CD206 protein expression of similar intensity that was attenuated by anti-HuR ASO was also detected in Western blotting experiments performed on spinal cord lysates (Fig. 6D). No significant variation in the expression of the anti-inflammatory markers IL-10 was detected on spinal cord lysates from EAE mice, and the cytokine level remained unmodified following anti-HuR administration (Fig. 6E). These results suggest that in the PLP-induced EAE model, anti-HuR ASO switched activated microglia cells from a proinflammatory to anti-inflammatory phenotype. This effect was mainly related to an increased expression of CD206-expressing cells rather than to the promotion of IL-10 anti-inflammatory cytokine release.

### Anti-HuR ASO Reduced EAE Clinical Disease and Locomotor Disability

To evaluate whether the reduction of glial cell activation induced by HuR silencing provides positive effects on EAE-related symptoms in mice, we examined the effect of anti-HuR ASO treatment on motor disability. ASO effects were compared to those produced by dODN administration, used as control ODN, and no behavioral alteration was observed in comparison with vehicle- or saline-treated mice, ruling out the presence of sequence-independent effects (Supplemental Fig. S1).

**Fig. 7:**
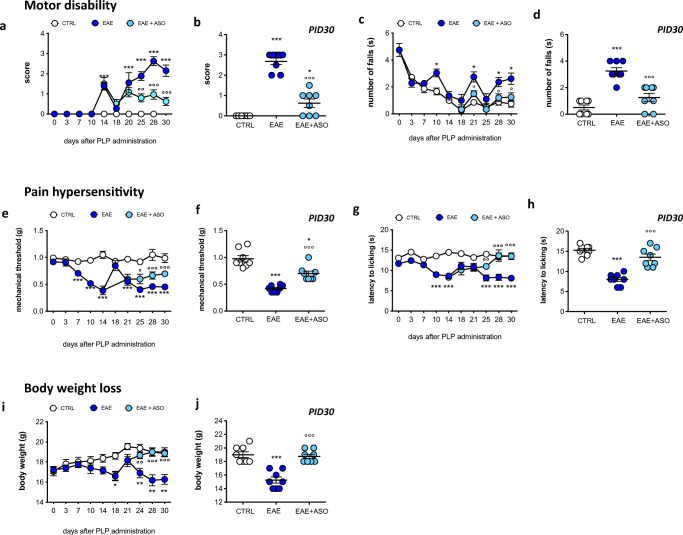
Anti-HuR ASO alleviated neurological disturbances in EAE mice. (a) Clinical disease score of PLP_139–151_-EAE mice compared to control mice (CTRL) showing a relapsing–remitting profile. Attenuation by HuR silencing of the increased disease clinical EAE score. (b) Scatter plot graph for anti-HuR ASO effect on day 30 postimmunization. *p < 0.05, ***p < 0.001 *versus* CTRL mice (Wilcoxon test); °°°*p* < 0.01 *versus* EAE mice (Kruskal–Wallis). (c) Progression of locomotor impairment in EAE mice by evaluating the rotarod performance in comparison with CTRL mice. Knockdown of HuR protein attenuated rotarod impairment over time. (d) Scatter plot graph for ASO effect on postimmunization day 30 (PID30). (e) Time course study of mechanical allodynia in EAE mice showing a relapsing–remitting profile. HuR silencing attenuated mechanical allodynia. (f) Scatter plot graph for ASO effect on postimmunization day 30 (PID30). (g) Time course evaluation of thermal hyperalgesia in EAE mice. Anti-HuR ASO alleviated thermal hypersensitivity. (h) Scatter plot graph for anti-HuR effect on postimmunization day 30 (PID30). (i) Time course evaluation of body weight loss of EAE mice in comparison with CTRL mice. Anti-HuR ASO reduced the body weight loss. (j) Scatter plot graph for anti-HuR effect on day 30 postimmunization. ASO-induced effects were evaluated starting from postimmunization day 14 (first day of ASO treatment). CTRL, dODN-treated nonimmunized mice; EAE, dODN-treated EAE mice; EAE + anti-HuR, anti-HuR ASO-treated EAE mice. *p < 0.05, **p < 0.01, ***p < 0.001 *versus* CTRL mice; °*p* < 0.05, °°*p* < 0.01, °°°*p* < 0.01 *versus* EAE mice (2-way ANOVA)

Time course experiments showed that PLP-EAE mice experienced motor disability with a relapsing–remitting disease profile. The clinical score evaluation revealed a first disease peak on day 14, followed by a remission phase. From day 20, a relapse phase appeared, and motor disability increased up to day 28 (Fig. 7A). We additionally performed an objective measure of locomotor disability on EAE mice by using the rotarod test to avoid a potential observer bias in scoring disability in EAE scores, being a subjective evaluation. Consistent with clinical score results, the rotarod performance, tested from day 3 up to day 30 postimmunization, was characterized by a relapsing–remitting disease profile with increased the number of falls during relapses (Fig. 7C). Time course experiments showed that HuR silencing drastically reduced locomotor impairment as demonstrated by the significant decrease in the clinical score values (Fig. 7A, B) and number of falls on day 30 (Fig. 7C, D).

### HuR Silencing Attenuated EAE-Associated Pain Hypersensitivity in the Hind Paws

In EAE mice, reactive gliosis and neuroinflammation in the spinal cord develop concurrently with robust neuropathic pain behaviors [35]. We, thus, investigated whether reduction of reactive microglia was coincident with a reduction of pain hypersensitivity by evaluation of the nociceptive thresholds in response to heat and mechanical stimuli. A significant reduction of the mechanical threshold in the bilateral hind paws was observed from day 7 that fully developed on day 14. EAE mice developed a second nociceptive mechanical allodynia peak on day 21 which persisted until study completion (Fig. 7E). Time course studies showed a relapsing–remitting nociceptive profile also for thermal hyperalgesia. The response latency towards heat stimuli dropped significantly in EAE mice on days 10–14. Mice developed a second thermal hyperalgesia peak on day 25 postimmunization which persisted until the end of the study (Fig. 7G).

HuR silencing attenuated the EAE-induced mechanical allodynia (Fig. 7E, F) and drastically reduced thermal hyperalgesia (Fig. 7G, H) over time. HuR silencing did not modify the animals’ pain threshold in nonimmunized control mice. dODN administration did not produce any alteration of pain hypersensitivity in comparison with vehicle- or saline-treated mice.

### Anti-HuR ASO Improves General Health and Body Weight Loss

EAE mice showed a progressive reduction of body weight that peaked on day 14. A second peak of reduction in the body weights was detected on day 21 postimmunization which persisted up to the end of the experiment (Fig. 7I). Time course experiments showed that treatment with the anti-HuR ASO counteracted the body weight loss observed in EAE mice (Fig. 7I, J) and improved the general health of immunized mice. dODN did not influence body weight values in comparison with vehicle- or saline-treated mice.

## Discussion

The present study aimed to elucidate the role of post-transcriptional regulation of gene expression in the EAE-related neuroinflammation by investigating the role of the RBP HuR, a member of the ELAV family. As the majority of MS patients manifest a relapsing–remitting form of MS (RRMS) [36], the study was conducted in a model of EAE obtained by immunization of SJL female mice with PLP_139–151_, recognized as an animal model of RRMS [37].

Activation of CNS-resident glial cells is involved in neuroinflammation, a main feature of MS pathophysiology. Mounting evidence has linked microglia activation to disease progression. Activated microglia can phagocytose myelin, leading to myelin damage, and produce inflammatory mediators which exert detrimental effects on MS [38]. A large increase of microglia immunostaining was detected in spinal cord sections of EAE mice and anti-HuR ASO normalized EAE-associated increase of activated microglia. Double immunofluorescence images showed a large degree of colocalization of HuR with CD11b, a marker of activated microglia, indicating these cells as a possible site of HuR action. H&E staining of spinal cord sections from EAE mice showed the presence of inflammatory infiltrates whose appearance was largely reduced by HuR silencing. Consistent with the hypothesis of a role of glial cells in neuroinflammation, these data indicate that microglia activation was concomitant to a proinflammatory response that involved a HuR-mediated mechanism.

Activation of the NF-κB pathway represents the molecular driver of proinflammatory microglia, found in MS brain tissue, where it localizes to astrocytes, oligodendrocytes, microglia, and infiltrating macrophages in or near CNS lesions [39, 40]. Activation of NF-κB often results in an inflammatory environment that propagates demyelination and inhibits remyelination in the CNS [30]. We observed a progressive increase in the activation of the NF-κB pathway in the spinal cord of PLP_139–151_-EAE mice. Consistent with results showing a HuR-mediated reduction of activated microglia, NF-κB activation was counteracted by anti-HuR ASO. Double immunofluorescence experiments showed the expression of HuR in the same cell population that expresses NF-κB signaling components, further supporting a functional interaction between HuR overexpression and NF-κB activation in EAE mice.

An important effector function of activated microglia is the secretion of proinflammatory cytokines, such as IL-6, IL-1β, IL-23, and TNF [41], that play an important role in both establishing and maintaining EAE and MS by regulating cell migration, proliferation, and activation of resident and infiltrating cells [42]. An increased expression of TNF-α and IL-1β protein levels was detected both in the spinal cord and plasma of PLP-immunized mice. Consistent with the cytokine mRNA stabilizing activity of HuR [12, 13, 43], anti-HuR ASO administration reduced the levels of the investigated cytokines, indicating the important role played by HuR in the proinflammatory response in the PLP_139–151_-EAE model.

Among cytokines involved in the pathogenesis of MS and EAE, a major contribution of IL-17 has emerged in the last decades. High levels of IL-17 have been detected in both plaques and CSF of patients with MS, and the high expression of IL-17 correlates with MS severity [44, 45]. The absence of IL-23, a Th17-promoting cytokine, made IL-23^−/−^ mice very resistant to EAE [46]. HuR silencing drastically reduced the increased IL-17 levels both in the spinal cord and plasma. Because IL-17 has been shown to promote neuroinflammation in various EAE models through the induction of proinflammatory cytokines, such as TNF-α, IL-6, and IL-1β [47], we cannot exclude that the reduction of TNF-α and IL-1β produced by HuR silencing can also be indirectly sustained by the decrease of IL-17.

MS is characterized by a compromised BBB, infiltration of macrophages, T cells, and B cells into the CNS, and local microglia and astrocyte activation, together promoting inflammation, demyelination, and neurodegeneration [42]. The BBB disruption is an early and central event in MS pathogenesis, and we demonstrated that anti-HuR ASO largely reduced breakdown of BBB in the brain and spinal cord during EAE. Recent studies revealed this event underlies an IL-17-mediated mechanism. The structure of the BBB includes vascular endothelial cells that are strongly joined by tight junctions [48]. Endothelial cells express IL-17 receptor, and IL-17 destroys the tight junctions of the BBB to facilitate the migration of CD4^+^ T cells in humans and mice [49, 50]. Because HuR can directly bind to the IL-17 mRNA, thus regulating IL-17 expression [10], IL-17 can be hypothesized as a prominent target of anti-HuR ASO in attenuating BBB compromise. To correlate cellular and molecular data to a positive effect on EAE-related symptoms, we evaluated the behavioral effects produced by anti-HuR ASO treatment on the clinical outcomes of EAE. In agreement with our previous study [51], HuR silencing reduced severity scores for motor disability, attenuated body weight loss, and ameliorated pain hypersensitivity, standard outcome measures for EAE. These findings show a functional correlation between the attenuation of neuroinflammation and an improved behavioral phenotype, further straightening the role of HuR in EAE progression and maintenance.

Microglia display remarkable plasticity in their activation phenotypes and present different activation states with the stage of MS and EAE disease. In the early stages, microglia are expressed as the proinflammatory phenotype [52]. The proinflammatory activated microglia were considered to be deleterious in MS by releasing destructive proinflammatory mediators and were involved in the development and expansion of lesions [5]. In the later stage, the anti-inflammatory phenotype becomes predominant in the CNS [53] to dampen neuroinflammation and help diminish neuronal damage [54]. This evidence leads to the hypothesis of an important role in disease progression played by proinflammatory/anti-inflammatory phenotype balance. Different anti-inflammatory microglia subtypes with different activation and functional mechanisms have been described, including a subtype with a regenerative phenotype (CD206+) and a subtype with a wound healing phenotype (CD163+, IL-10+) [55]. In the spinal cord of EAE mice, we detected a significant decrease of CD206 expression in the absence of any alteration in the IL-10 content. Anti-HuR ASO treatment restored CD206 expression whereas the IL-10 levels remained modified, consistently with the observation that IL-10 inhibits the mRNA expression of proinflammatory cytokine/chemokine in human monocytic cell line and in primary mouse macrophages by inhibiting HuR [56]. These findings indicate that HuR silencing, in addition to the attenuation of proinflammatory events, promotes a shift to the anti-inflammatory and regenerative anti-inflammatory microglia phenotype. Even though anti-inflammatory-induced dampening of neuroinflammation is also correlated to promoting release of anti-inflammatory cytokines [57], present findings indicate that HuR prominently influences regenerative CD206+ anti-inflammatory microglia cell functioning rather than modulating anti-inflammatory cytokine expression.

Increasing evidence shows proremyelinating activities following anti-inflammatory microglia activation. CD206-expressing microglia are abundant in MS active lesions, in which remyelination occurs, and are almost absent in lesions where remyelination is poor [17]. CD206-expressing anti-inflammatory microglia can also inhibit proinflammatory activation by suppressing the NF-κB signaling pathway contributing to protect neurons from damage [58]. Thus, manipulating transition from proinflammatory to CD206+ anti-inflammatory microglia phenotype might represent a desirable strategy for efficient remyelination therapies. Our previous data reported an attenuation by HuR knockdown of the decrease of Luxol Fast Blue staining, indicative of significant demyelination, in the spinal cord of EAE mice [59], suggesting a possible role of HuR on remyelinating processes in EAE.

Due to its nature of RNA-binding protein, HuR can modulate simultaneously mRNAs involved in inflammatory and regenerative phenomena, representing an innovative and promising target. Available MS therapies, which halt the inflammatory and immune response, do not always stop disease progression and cumulative neurological disability, and none of the current treatments halts or cures the disease [3]. Thus, evidence suggests that treatment of MS should be based on a combination of anti-inflammatory, regenerative, and neuroprotective strategies [60]. Our current findings show that anti-HuR ASO reduced spinal inflammatory infiltrates, proinflammatory cytokine levels, BBB disruption, and proinflammatory microglia activation; promoted the activation of CD206-expressing anti-inflammatory microglia; and alleviated the severity of EAE clinical signs. Many similarities between EAE and MS have been described, including clinical course, pathological CNS lesions, glial activation, and axonal demyelination [61]. Thus, it is plausible that targeting HuR might represent a promising perspective to control neurological disturbances in MS patients.

## Supplementary Information

ESM 1 (PDF 1224 kb)

ESM 2 (PPTX 151 kb)
