# Supplementary material for: Targeting the RNA-Binding Protein HuR Alleviates Neuroinflammation in Experimental Autoimmune Encephalomyelitis: Potential Therapy for Multiple Sclerosis
Source: Neurotherapeutics. 2020 Nov 16;18(1):412–29. doi: 10.1007/s13311-020-00958-8 (PMC8116432; doi:10.1007/s13311-020-00958-8)
Supplement: Supplementary file 2 — (PPTX 151 kb) [file 13311_2020_958_MOESM2_ESM.pptx]

## Slide 1
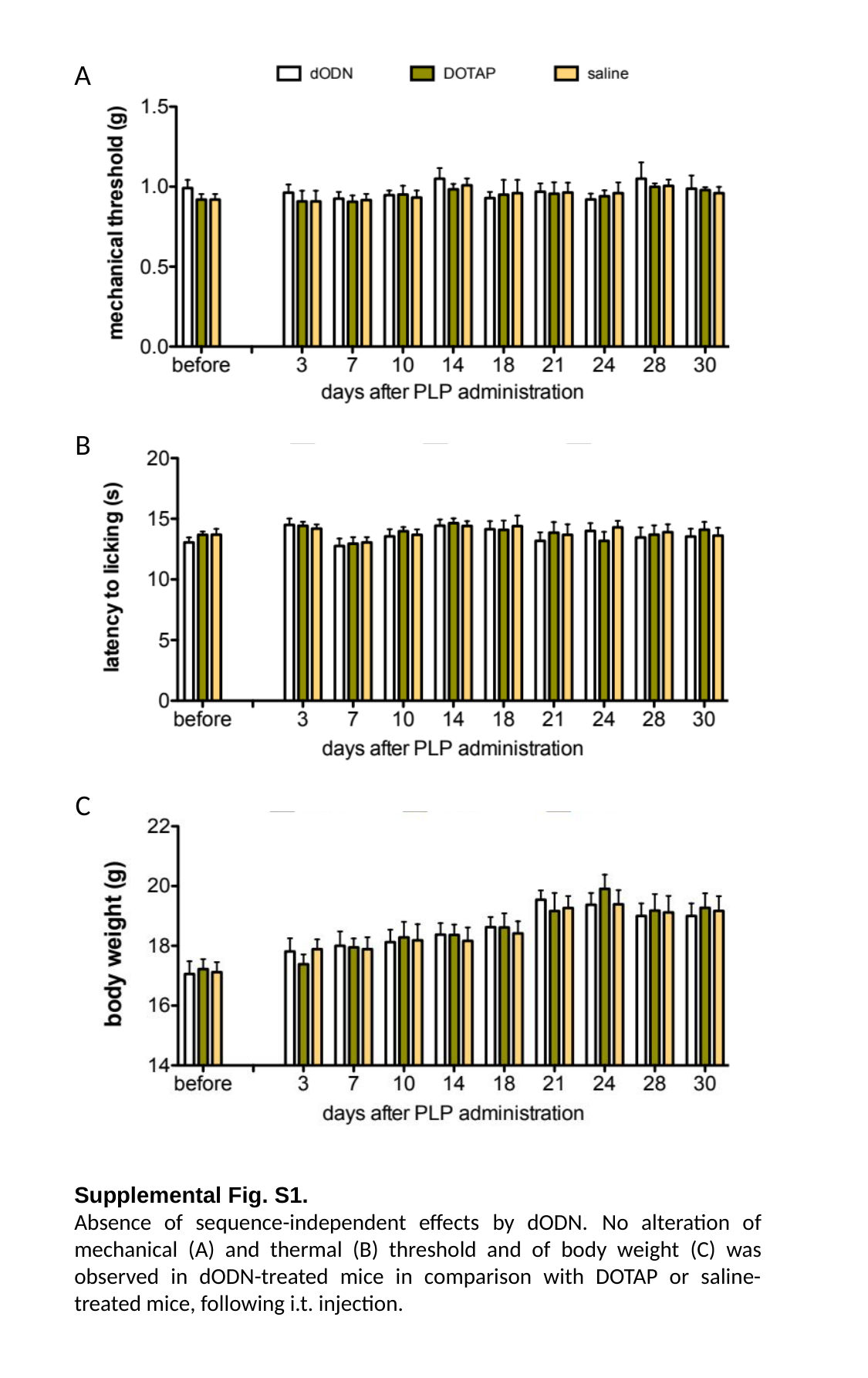

A
B
C
Supplemental Fig. S1.
Absence of sequence-independent effects by dODN. No alteration of mechanical (A) and thermal (B) threshold and of body weight (C) was observed in dODN-treated mice in comparison with DOTAP or saline-treated mice, following i.t. injection.
